# An Update on Molecular Biology of Cutaneous T Cell Lymphoma

**DOI:** 10.3389/fonc.2019.01558

**Published:** 2020-01-22

**Authors:** Ritika Walia, Cecilia C. S. Yeung

**Affiliations:** ^1^Clinical Research Division, Fred Hutchinson Cancer Research Center, Seattle, WA, United States; ^2^Department of Pathology, University of Washington, Seattle, WA, United States

**Keywords:** cutaneous T cell lymphoma (CTCL), molecular biology, TCR clonality, mycosis fungoides, NGS, single cell sequencing

## Abstract

Cutaneous T cell lymphomas represent a heterogenous group of lymphoproliferative disorders defined by clonal proliferation of T cells present in the skin. The latest WHO classification in 2016 and WHO-EORTC classification in 2018 has updated the classification of these entities based on the molecular profile. Research in the field of molecular genetics of CTCL has allowed a better understanding of the biology of these tumors and has helped to identify potential targets for therapy that can be tailored to individual patients. In this review, we discuss the latest developments in the molecular profile of CTCLs including biomarkers for diagnosis, prognosis, and potential therapeutic targets. We have also touched upon the utility of various molecular diagnostic modalities. For the purpose of this review, we researched papers in PubMed indexed journals in English literature published in the past 20 years using keywords CTCL, mycosis fungoides, molecular profile, molecular diagnosis, whole genome profile, genomic landscape, TCR clonality.

## Introduction

Cutaneous T cell lymphomas (CTCL) are a heterogenous group of lymphoproliferative disorders arising primarily in the skin without the evidence of extracutaneous involvement. For the purposes of this review, we define CTCL according to the WHO 2016 and the 2018 WHO-EORTC classification system. Since, the 2008 WHO classification, molecular advances with gene expression profiling and a better understanding of the genomic landscape of TCL allowed for improved classification. Specific phenotypes such as the T follicular helper cells, are now possible to identify with gene expression profiling. In the 2016 WHO and 2018 WHO-EORTC classification of skin tumors a few provisional entities have been added, like chronic EBV positive mucocutaneous ulcer, primary cutaneous acral CD8+ T cell lymphoma, Primary cutaneous CD4+ small/medium lymphoproliferative disorder. Three distinct types of lymphomatoid papulosis in addition to originally described types A,B,C have also been included (1, 2).

In this review, we discuss the molecular genetic profile of CTCL's along with new molecular biomarkers of diagnostic and prognostic significance with respect to the latest classification.

## Molecular Profile Of CTCLs

Mycosis Fungoides/Sezary syndrome (MF/SS) comprise the majority of cutaneous T cell lymphomas, most of the genomic studies elucidating the pathogenesis and genetic abnormalities being studied in this group of CTCL. There are no disease specific molecular abnormalities that define these lymphomas, however some changes are more frequent than others.

Whole genomic sequencing studies of MF/SS have revealed somatic mutations in genes involved in TCR/NFκB signaling, Th2 differentiation, cell survival and fate, epigenetic regulation, homologous recombination, and cell-cycle control. These pathways in the pathogenesis of CTCL have opened new avenues for prognostication, disease monitoring, and targeted therapy (3, 4).

Recurrent mutations identified by whole exome/genome sequencing in common oncogenic pathways include- DNA damage repair (i.e., *ATM, TP53*), cell cycle (i.e., *CDKN2A, RB1*), apoptosis (i.e., *FAS, TNFRSF10A*), MAPK pathway (i.e., *KRAS, BRAF, MAPK1*), and chromatin modifying genes (i.e., *ARID1A, DNMT3A, KMT2C*). Forty to ninety two percentage of these mutations are single copy number variations (SCNVs) (3, 5–7). Increased burden of somatic single nucleotide variations (SSNVs) have also been identified in various genes like *Tp53, RHOA*, CD28*, DNM3TA*. Interestingly, SCNV's are more important mutational drivers for CTCLs than SSNV's when compared to other cancers with significantly higher SCNV/SSNV ratios (5). Increased number of transition (C-T or G-A) mutations are observed in 40–60% of cases along with a higher frequency of dinucleotide variations indicating that ultraviolet (UV) radiation exposure may have a role in the pathogenesis of CTCL (4, 8).

Alterations in T cell signaling pathways have also been described in recent studies which include mutations in co-stimulatory molecules (CD28 and CTLA4), TCR associated enzymes (*PLCG1*) as well as downstream transcriptional regulators of TCR function (*ZEB1*) (4, 5, 8). Out of these, a novel *CD28-CTLA4* gene fusion (6) resulting in acceleration of TCR signaling has been exploited for anti-CTLA4 therapy (ipilimumab) with promising results (9). Recurrent point mutations and deletions in *ZEB1*, a zinc-finger transcription repressor, have been found in 56–65% of CTCLs. This functional inactivation of *ZEB1* leads to overproduction of TH2 cytokines promoting tumor growth in CTCLs (4, 5, 8). *PDCD1*, a gene on chromosome 2p expressing PDL1 has also been found to be deleted in 36% of cases. *PDL1* is found on activated T cells and gives a negative signal to suppress the T-cell function. This provides a rationale for use of anti PDL1 therapy for CTCL as well.

NFκB signaling pathway has been shown to be affected in cutaneous lymphomas by several mutations leading to its constitutive activation (6, 10) Recurrent point mutation (Thr377Ile) of *TNFRSF1B* found in 18% MF cases is one such example. NFκB is a nuclear transcription factor regulating gene expression of various growth promoting factors like TNFα, IL-2, IL-6, TGFβ, IFNβ. NFκB is normally sequestered in the cytoplasm by IκB, and it can translocate to the nucleus only when IκB is ubiquitinated or degraded by proteosomes. Recurrent deletions of C-terminus of NFκB leads to proteosomal cleavage of IκB causing constitutive activation (5). These mutations make these tumors amenable to proteosome inhibitors like Bortezomib (11). *CARD11* potentiates NFkB signaling in T and B cells has been found to be mutated in a subset of SS cases and has been suggested as a potential therapeutic target as in DLBCL (8).

Activating mutations in JAK/STAT pathway including *JAK1, JAK3, STAT3*, and *STAT5B* were found in a subset of cases by many groups. Anti-tumor properties of *JAK1/2* inhibitor Ruxolitinib and *JAK 1/3* inhibitor Tofacitinib have been tested in CTCL cell lines with promising results (3–5, 12). Two tumor suppressor genes (*HNRNPK* and *SOCS1*) which are inhibitors of JAK-STAT signaling have been identified to be recurrently deleted in MF also represent potential therapeutic targets (13).

## T-Cell Receptors And Clonality Testing: Diagnostic Utility

T cell development begins in the thymus, where previously uncommitted progenitors migrate from the marrow to become double negative thymocytes and start rearrangement of their T cell receptor (TCR) genes (TCRα, TCRβ, TCRγ, TCRδ). The TCR is a heterodimer of either alpha/beta or gamma/delta type. Each T cell and its progeny have a unique rearrangement of TCR genes formed by diverse recombination of V/J or V/D/J segments during T cell development (14). It is widely accepted that T cell malignancy arise from a single T cell clone, hence testing of malignant cells will demonstrate the same TCR gene sequence, termed as monoclonal. Monoclonality can also be seen in autoimmune and reactive conditions (15, 16) and some malignancies can also have oligoclonal populations. Therefore, assessment of clonality should be performed in appropriate clinico-pathological scenario. Molecular methods used are described below briefly.

CTCL's are clonal disorders arising from skin homing mature T cells showing specific TCR gene rearrangement. CTCL's are routinely diagnosed by clinical presentation, histopathology and immunohistochemistry of the atypical infiltrate with clonality assays serving only as adjuncts to the diagnosis. Comfere et al. reported that only 2.3% dermatologists actually order TCR clonality assays as part of the initial diagnostic evaluation and only 3.7% of pathologists rely on them for diagnosis of cutaneous lymphoproliferative disorders (17).

Early lesions of CTCLs with lesser number of abnormal lymphoid cells typically pose a diagnostic challenge since various benign dermatoses have similar histologic features and immunohistochemistry is also not reliable in such cases (18, 19). Detection of TCR gamma and beta gene rearrangement can be of value in these scenarios as benign dermatitis does not show clonal rearrangement (19). However, the sensitivity of detecting TCR rearrangement by PCR is highly variable ranging from 50–90% in various studies (20–22). The reasons are multifactorial such as stage of disease, method of assay, primer design, PCR products and interobserver variability.

The success of PCR based assays in clonality assessment depends hugely on the choice of oligonucleotide primers and PCR design. This can be partly overcome by using standardized set of primers for e.g., BIOMED 2 (23, 24). Capillary gel electrophoresis was found to be better than conventional PAGE analysis to study the PCR products (25, 26).

Application of Next generation sequencing (NGS) /high throughput sequencing has greatly improved the sensitivity of detection (27–29). Sufficool et al. found a sensitivity of 85% using NGS as opposed to 44% using PCR. Table 1 summarizes various studies showing variable sensitivities of TCR clonality techniques.

**Table 1:** Summary of studies showing the sensitivity of various techniques for T cell clonality assessment in CTCL and benign inflammatory skin disorders.

**References**	**Technique used**	**Sensitivity in CTCL**	**BID cases showing monoclonality**	**Notes/Remarks**
Dippel et al. (30)	TCRg PCR-GS	76%	14%	Benign cases included psoriasis, eczema.
Schiller et al. (31)	TCRg PCR-DGGE	ND	25%	Lichen Planus
Lukowsky et al. (15)	TCRg PCR-HRE	ND	49%	Lichen sclerosus et atrophicus
Sandberg et al. (23)	SB TCRg PCR (GS and HD) TCRb PCR	68% 76% 66%	ND	Used BIOMED-2 protocol
Ponti et al. (16)	TCRg PCR-HD	83.5%	2.3%	
Hsiao et al. (21)	TCRg PCR	53%- Patch 100%- Plaque 100%-Tumor	ND	PCR not very sensitive in early stages
Tang et al. (26)	TCRg PCR FCE PAGE	77.3% 63.6%	ND	FCE is a better method of detection of PCR products
Lukowsky et al. (24)	TCRg PCR TCRb PCR Combined sensitivity	81% 78% 87%	ND	Biomed-2 protocol was used
Sufficool et al. (28)	TCRg PCR-CE NGS	44% 85%	ND	
Kirsch et al. (27)	TCRg PCR NGS	70% 100%		Patients with negative results by PCR had early stage disease
Rea et al. (29)	TCrg PCR NGS/HTS	72% 68%	ND	HTS was more specific than PCR, 100% vs. 88%.

*TCRg/b, T cell receptor gamma/beta; PCR, Polymerase Chain Reaction; GS, Gene Scan; HD, Heteroduplex; HRE, High Resolution Electrophoresis; FCE, Fluorescent Capillary Electrophoresis; SB, Southern Blot; DGGE, Denaturing Gradient Gel Electrophoresis; PAGE, Polyacrylamide Gel Electrophoresis; ND, Not Done; NGS, Next Generation Sequencing; HTS, High Throughput Sequencing; BID, Benign Inflammatory Dermatoses*.

T-cell clonality is itself not diagnostic of a T cell lymphoma as many dermatitis may have dominant T cell clone (16, 18, 30, 31) and 25% of these clonal dermatitis eventually evolve into CTCL (14). Identification of a single clone at two different anatomic sites favors a diagnosis of a malignant process over benign (32). On the contrary, the finding of polyclonality/oligoclonality does not rule out the possibility of CTCL. Therefore, the diagnosis of lymphoma should be made in conjunction with clinical and histopathological features using TCR clonality as an adjunct (23).

TCR gene rearrangement can be utilized for monitoring of residual disease particularly in leukemic involvement by MF/SS. Standard methods used to accomplish this are through flow cytometry using tumor specific TCR Vbeta antibodies (33) or TCR PCR which is more sensitive and specific (34). The monoclonal peak in the original sample is identified and followed up with repeat samplings. This monoclonal peak is however not always tumor-specific as few non-malignant T cell clones may give rise to the same. A large 2015 French study on over 200 patients with MF showed no significant prognostic differences at 4 years of clinical follow-up when MRD was determined by TCR clonality based on PCR studies (35), however a similar study utilizing NGS has not been performed. Weng et al. employed NGS/HTS technique for MRD detection in MF/SS and showed it to be highly sensitive and specific for detecting disease however their study was small (*n* = 10) and did not have long term outcomes data (<2 years) (36). This is specifically useful in post stem cell transplant recipients where a skin rash could represent drug toxicity, GVHD or recurrence and clinical and pathological distinction can be quite challenging (37, 38). NGS of TCRB can be of value in such critical decision making scenarios (36).

Identification and monitoring of monoclonality, however, seems to have no prognostic relevance even if identified in early lesions (35, 39) However, tumor clone frequency (TCF) obtained by HTS of TCRb gene is a strong and independent prognostic marker for progression free and overall survival in CTCL-MF. TCF > 25% at an early stage of MF has the ability to predict a poorer outcome than any other prognostic marker (40).

## Molecular Diagnostic Modalities For TCL

### Clinically Used Techniques

#### PCR Based Assays

PCR of the TCR gamma and/or beta gene is frequently used as an adjunct to asses monoclonality in T cell lymphomas. TCRG PCR is preferred as gamma gene is rearranged earlier and present in most of the T cells and has only 12 segments, hence less primers are to be used (41). Combined use of TCRB and TCRG primers increases the sensitivity of the test than using each of them individually (24, 42). The extracted DNA from fresh tumor, liquid samples, or formalin fixed paraffin embedded tissue specimen can be tested using PCR amplified with commercially available primers. The PCR products are analyzed by capillary electrophoresis or genescan depending upon the size of the amplicon. In straightforward cases, a single dominant peak is seen if the infiltrate is monoclonal whereas multiple peaks when it is polyclonal (43). However, in clinical practice challenging cases and clinical scenarios occur and a good understanding of the starting sample, patient history as well as the specific assay parameters are important for the molecular pathologist in their interpretation of results. This analysis is subjective and liable to inter-observer variability since it is based on qualitative assessment of relative peak heights. Many cases do not have a dominant clone while a dominant peak may represent a mixture of clones of similar size (44). Therefore, it is recommended to run the PCR in duplicate, and only reproducible peaks should be considered positive (41).

Molecular testing for infectious agents that are seen in some CTCLs are mostly PCR based. Input samples may include testing of peripheral blood or tumor tissues. However, understanding the pathobiology of how the infectious agent leads to tumorigenesis is important in diagnosis. One example include Adult T-cell leukemia/lymphoma, a monoclonal integration site of HTLV1 virus genome into the host cell leads to upregulation of *HBZ* in most cases impacting cell growth, immune response and differentiation.

#### Next Generation Sequencing Based Assays

NGS has vastly improved TCR clonality assessment mainly through improved specificity via sequencing of rearranged TCR. Published evidenced is increasing for the use of this technology in determining T cell clonality status (27–29, 44) In this technique, TCRG and TCRB loci are PCR amplified using consensus primers, PCR products are purified and made into libraries and ligated to adapters which are then sequenced by synthesis using labeled nucleotides. The malignant clone is identified by sequence abundance. If most frequent two sequences consist of the predominant sequence, the population is called clonal (28). This technique has advantage of being quantitative, robust and reproducible. NGS has successfully proven to be useful for early detection of CTCL as well in differentiating malignant from benign lesions especially where PCR is negative (27, 44). A clonal sequence can be identified in as low as 1 in 50,000 malignant cells when flowcytometry is not able to reveal residual disease (36). Patients in molecular remission can thus be reliably identified using NGS. Minimal residual disease (MRD) detection by NGS have been described to be reliable with some clinically validated platforms for use in T cell lymphomas such as sezary syndrome (36) and other CTCLs (27).

### Molecular Techniques Being Used for Research Domain

#### Single Cell Sequencing

Although this technology is still relatively contained within the research space, clinical applications using this technology will likely advance patient care in the next decade. Single cell sequencing studies have shown evidence of tumor heterogeneity in CTCLs and there is also preliminary evidence that there may be a prognostic impact (45–47). Demonstration of specific gene expression profiles enabling examination of the tumor microenvironment along with the tumor cells in the same assay provides useful insights to tumor biology and immune response, which ultimately helps in tailoring personalized treatments, understanding prognosis, response to therapy, and reasons for drug resistance (48, 49).

Gaydosik et al. performed single cell RNA sequencing (scRNA) using droplet based sequencing to study thousands of individual cells in advanced CTCL's along with skin samples from healthy controls. The results not only revealed significant inter-tumor but also intra-tumor heterogeneity. Many CTCL's had differential expression of genes that was unique to that tumor signifying its importance in personalizing patient specific therapy. A common gene expression profile of highly proliferating lymphocytes was identified which was present in all tumors indicating its potential diagnostic role (47).

Buus et al. demonstrated distinct subpopulations of tumor cells in Sezary syndrome (SS) through ScRNA sequencing and flow cytometry while studying drug resistance to histone deacetylator inhibitor. Some cell populations were responsive to the drug while others were resistant. This study sheds light on the utility of this technique in its therapeutic implications (45).

Similarly, Borcherding et al. described the transcritpomic diversity in Sezary cells using ScRNA sequencing can be used to predict disease stage and guide therapy (46).

#### Gene Expression Profiling

In the past decade, gene expression profiling have been used to identify unique T cell lymphoma subtypes leading to new provisional diagnostic entities. The molecular platforms include mostly research use only, however increasingly clinical grade assays are becoming available. The earliest and possibly most widely available are gene expression microarrays (50). These assays are robust with well-established analysis tools and pipelines but time consuming and require highly skilled staff. Newer platforms that enable direct capture and counting of specific RNA molecules via a barcode or color-coded bead combinations have improved on prior generations of DNA probe based gene expression arrays (51). Gene expression by RNA sequencing is yet another platform that is based on NGS technology, where instead of sequencing input sample of DNA, RNA is used and therefore an expression profile is generated based on the relative numbers of cDNA molecules that are created in the NGS library preparation. A recent study compared RNA sequencing results from 181 fresh and FFPE CTCL or inflammatory dermatoses skin tissues and showed that specific genes were upregulated in benign skin conditions or low grade CTCL and that a gene signature could be applied to differentiate patients with more aggressive disease (52). In addition to RNA gene expression profiling, microRNA profiling is also possible with the technologies described in this section. More detailed description of specific expression profiles are described in the disease entities below.

## Disease Entities: Molecular Markers Of Diagnostic, Prognostic, And Therapeutic Significance

### Mycosis Fungoides/Sezary Syndrome (MF/SS)

Mycosis fungoides is the most common form of CTCL encompassing 50–60% of all CTCLs (53). Sezary syndrome (SS) is the leukemic variant presenting with erythrodermic lesions along with lymph node and peripheral blood involvement at presentation. MF is skin limited and leukemic involvement occurs only in few cases progressing to advanced disease. MF/SS are considered distinct entities but included in the same staging system (34, 53). MF and SS arise from different types of CD4+ memory T cell. MF T-cells are T resident memory (Trm) cells exhibiting CCR4+/CLA+/ L-selectin-/CCR7– (TRM), whereas SS malignant T-cell are central memory cells (Tcm) (CCR4+/L-selectin+/CCR7+) (54). Trm cells are skin tropic and stay within the epithelial barriers while Tcm cells have the ability to shuffle between skin, lymph nodes and blood (55), which provides an understanding of some clinical differences between MF and SS.

MF passes through various clinical stages and early lesions (eMF) posing a significant diagnostic challenge when differentiating from benign dermatoses (18). The utility of T cell clonality studies, discussed above, can be useful but must be interpreted with caution with full understanding of the clinical and pathologic picture. High throughput sequencing of TCR CDR3 region can identify a smaller clone of malignant cells in comparison to PCR which may miss a low level clonal process (27).

Gene expression profiling of MF/SS has elucidated a diagnostic panel of genes that can reliably characterize them. Litvinov et al. describes a 17 gene signature including IL2RA, CCR4, STAT5A, and TOX that is able to identify patients who are at risk of progression and differentiate MF/SS from benign dermatoses (56). Nebozhyn et al. proposed a panel of five genes (*STAT4, GATA3, PLS3, CD1D*, and *TRAIL*), Michel et al. proposed a panel of 4 genes (*PLS3, Twist1, CD158k/KIR3DL2*, and *NKp46*) using qRTPCR showing the ability to diagnose 91 and 100% cases of SS, respectively (57, 58). Amongst these *Twist1* alone has the power to diagnose 91% SS cases. *CD158k/KIR3DL2* has emerged as a therapeutic target with phase 1 clinical trials currently underway (52). *CD158k/KIR3DL2* is highly expressed in SS and advanced MF confers resistance to activation induced cell death in SS and has been found efficacious in reducing tumor size and improved survival *in vivo* and *in vitro* (59, 60).

Expression of CCR4 by the malignant T cells in CTCL has been exploited for targeted therapy by the use of anti-CCR4 monoclonal antibody mogamulizumab. This drug has recently been approved by US-FDA for treatment of relapsed or refractory MF and SS after phase 3 multicentric clinical trial (MAVORIC study) showed a significant improvement in progression free survival as compared to conventional treatment by acetone deacetylase inhibitor vorinostat (61). Granulomatous drug eruption is a frequent side effect of mogamulizumab which can be confused with disease progression but additional studies showed this side effect might represent adequate treatment response (62).

*TOX*, a gene that encodes a member of homeobox family and regulate T cell development in the thymus, is upregulated in early as well as advanced MF. Presence of *TOX* mRNA can help differentiate eMF from benign cases (63). Moreover, high expression of TOX mRNA in eMF is also strongly correlated with disease progression potentially allowing for intensifying treatment at an early stage for patients (64, 65). Some of the molecular markers and aberrations have already been discussed in the above paragraphs and hence, will not be discussed again.

Lastly, microRNA profiling has been extensively performed to evaluate its role in the diagnosis and management of MF/SS. Invaluable diagnostic potential with 95% sensitivity and specificity in differentiating eMF from benign lesions of a panel of three microRNAs namely miR-155, miR-203, and miR-205 has been elucidated (66). Measuring their levels in plasma can be used to monitor tumor burden and response to therapy (67). eMF and benign lesions display different miR profiles and as the disease progresses, different miRNAs become deregulated implicating their role in disease progression (68). 3-miRNA classifier, based on miR-106b-5p, miR-148a-3p, and miR-338-3p, can successfully segregate patients into high-risk and low-risk groups of disease progression (69).

All these studies confer that there is a wide variety of genomic alterations in MF/SS and the genetic signature of tumors can differ between patients. Hence, choosing personalized therapy for each patient is worthwhile according to the individual tumor's molecular pattern (70).

### Primary Cutaneous CD30+ T Cell Lymphoproliferative Disorders

The cell membrane protein, CD30 (aka TNFRSF8), is a member of tumor necrosis factor receptor family and is expressed on activated B and T cells as well as a number of lymphoid malignancies. These include a spectrum of disorders comprising of various types of lymphomatoid papulosis (LyP) and cutaneous Anaplastic large cell lymphoma (pcALCL). They are the second most common CTCL about 25% of all CTCLs (53). Clinical presentation is different in both LyP and pcALCL but they have overlapping pathological features (71).

The latest WHO-EORTC classification (2) has described new subtypes of LyP described types D- resembling primary cutaneous aggressive epidermotropic cytotoxic T-cell lymphoma (72); type E- angiocentric and angiodestructive and clinically characterized by large necrotic eschar-like lesions (73); LyP with presence of rearrangements of *DUSP-IRF4* locus on 6p25.3 (74). These are histological subtypes with little difference in the prognosis. There differentiation from other aggressive lymphomas is important.

#### LyP

Presence of detectable monoclonal T cell population varies in LyP and largely depend upon the number of T cells present in the lesion which is higher in type C than type A (75). Various studies have proved that MF or other lymphomas arising in patients with LyP have the same TCR rearrangement as the original lesion suggesting a clonal relationship between them (76). A recent study showed a high proportion of LyP have increased expression of SATB1 (91.7%) and that these cases were characterized by prominent epidermal hyperplasia and infiltration by polymorphs, with better response to methotrexate and interferon therapy (77). Although rare, rearrangement in the *DUSP22-IRF4* locus has led to a newly recognized subtype of LyP harboring a biphasic histological pattern with small-medium sized T cells exhibiting epidermotropism along with large T cells in the dermis, both endorsing CD30 positivity (78).

#### pcALCL

This is an indolent CD30+ lymphoma and involvement of extracutaneous sites needs to be ruled out because systemic ALCL portends a poor prognosis. There are various differences between pc ALCL and systemic ALCL on molecular level. Most cases of pcALCL are negative for classic chromosomal rearrangements seen in systemic ALCL—ALK, DUSP22/IRF4, and TP63 (79). DUSP/IRF4 rearrangement is found in 20% pcALCLs (78) and show a biphasic histological pattern similar to LyP with this alteration (80). There is no clinical or prognostic significance of this genetic change in pcALCL (81). *DUSP22/IRF4* is also not diagnostically specific for pcALCL as it has also been identified in few systemic ALCLs where is associated with better prognosis than *TP63* positive cases (82).

A novel *NPM1-TYK2* gene fusion has been identified in 2 cases (1LyP, 1pcALCL) of CD30+ LPDs by whole transcriptome sequencing. This chimeric protein activates STAT5 pathway and hence has been implicated as a therapeutic target (83). *TP63* rearrangement has also been described in two cases of pcALCL and both these cases had worse clinical progression like systemic ALCL (84).

ALK positivity by immunohistochemistry has been found in only a handful of cases of pcALCL and these cases showed a favorable prognosis (85) unlike their systemic counterpart. ALK translocation has not been found in pcALCL to date (79).

Most widely used targeted therapy is anti CD30 brentuximab in these neoplasms. Other potential therapeutic targets being developed are *KIR3DL2* (86), *NPM-TYK2*, and miR155 (87).

## Cuatneous Lymphomas With T Follicular Helper (TFH) Phenotype

Recent revision of WHO classification of hematolymphoid neoplasms has proposed a category of nodal T cell lymphomas wcell lymphoma with TFH phenotype and includes Angioimmunoblastic T cell lymphoma (AITL), Follicular T cell lymphoma (FTCL), and nodal peripheral T cell lymphoma (PTCL) with TFH phenotype. Diagnosis requires presence of atleast two markers of TFH lineage- CD279/PD-1, CD10, BCL6, CXCL13, ICOS, SLAM-associated protein (SAP), and CXCR5 (1).

Amongst these, AITL frequently involves skin in about 50% cases (88). TCR rearrangement was found in 82% cases (89).

*RHOA* p.G17v mutation has been found in 60–70% cases of AITL with equal frequencies in lymph nodes as well as skin (90, 91). This mutation is diagnostic of AITL and not present in other PTCL-NOS. Patients harboring this change also tend to have classic AITL histological features and an increased expression of TFH markers making it an important molecular marker of this lineage (92, 93).

Other mutations found in these cases by whole exome sequencing were *TET2, DNMT3A, IDH2* in various studies (94–96). Mutations in TCR related genes (*PLCG1*, CD28, *PI3K* elements, *CTNNB1*, and *GTF2I*) correlated with early disease progression (97). AITL is frequently infiltrated by atypical B cells which sometimes resemble Reed-Sternberg cells posing a diagnostic difficulty. Rearrangement of both Ig genes and TCR has been identified in some cases of AITL (98). When differential expression of genes was studied, *TET2* and *DNMT3A* mutations were found in B and T cells while *RHOA* and *IDH2* mutations were found only in tumor cells (99).

## Conclusion

Novel molecular discoveries have vastly advanced the field of CTCLs providing not only a deeper understanding of tumor biology, immune response, but also have introduced new targeted therapeutic strategies. Table 2 summarizes various molecular biomarkers being studied for targeted therapy in CTCL.

**Table 2:** Molecular biomarkers as therapeutic targets for CTCL.

**Molecular marker/mutation**	**Targeted therapy**	**Clinical status**	**References**
CD30	Brentuximab	Phase II trial	(100)
*CTLA4*	Ipilimumab		
*CD28-CTLA4* fusion	Ipilimumab		(9)
*CCR4*	Mogamulizumab	US-FDA approved	(61)
*TNFRSF1B*	Bortezomib	Phase II trial	(5, 6)
*NFkB*	Bortezomib	*In-vitro* studies, small phase II trials	(6, 101)
*CD158k/KIR3DL2*	IPH4102	Phase I trials	NCT02593045
JAK/STAT pathway	Ruxolitinib (JAK1/3) Tofacitinib (JAK1/2)	*In vitro* studies	(12)
*PDCD1*	Pembrolizumab	Phase II trial	NCT02243579)

Expert opinion: T cell clonality detection by NGS helps identify primary clonal sequences that is helpful for diagnosis of CTCL as well as follow up minimal residual analysis. TCR gamma PCR is the standard of care in most of the labs but it has a variable sensitivity and a lower specificity. In our opinion, NGS should be included in the diagnostic work up as well as management of MRD.

## Author Contributions

RW performed the literature search and wrote most parts of the manuscript. CY conceived the design and framework of the paper, wrote some parts, and edited the whole manuscript.

### Conflict of Interest

CY has research funding with OBI pharmaceutical and Pfizer for unrelated projects. The remaining author declares that the research was conducted in the absence of any commercial or financial relationships that could be construed as a potential conflict of interest.
